# Early Diagnosis Begets Timely Treatment: Multimodal Imaging Unraveling a Diffuse Amyloid Cardiomyopathy

**DOI:** 10.7759/cureus.79474

**Published:** 2025-02-22

**Authors:** Glenmore Lasam, Maria Kristina Cassandra Lasam, Loba Alam

**Affiliations:** 1 Cardiovascular Disease, Stockton Cardiology Medical Group, Manteca, USA; 2 Biomedical Science Pathway, Mountain House High School, Mountain House, USA; 3 Cardiovascular Disease, Icahn School of Medicine at Mount Sinai, New York, USA

**Keywords:** cardiac magnetic resonance imaging, multimodality cardiac imaging, myocardial biopsy, technetium-99m pyrophosphate scan, transthoracic echocardiogram, transthyretin amyloid cardiomyopathy

## Abstract

A case of a 67-year-old male with a history of hypertension, diabetes, and hyperlipidemia presented to the hospital because of chest discomfort, preceded by a gradual onset of diminished exercise tolerance, exertional dyspnea, orthopnea, paroxysmal nocturnal dyspnea, and bilateral lower extremity swelling. An electrocardiogram showed no acute ischemic changes, though he had very mild troponin elevation. Cardiac catheterization showed nonobstructive coronary artery disease. A transthoracic echocardiogram revealed reduced left ventricular systolic function with elevated left ventricular filling pressure. Cardiac magnetic resonance imaging demonstrated abnormal contrast kinetics, consistent with cardiac amyloidosis. A technetium-99m pyrophosphate scan unveiled diffuse cardiac amyloidosis of the transthyretin type. Myocardial biopsy showed amyloid deposition by positive Congo red stain and confirmed by apple-green birefringence on polarized light. He was started on medical therapy for heart failure and coronary artery disease. He was eventually started on an oral transthyretin stabilizer that significantly improved his heart failure symptoms and exercise capacity, with no readmission to the hospital since then. He had been evaluated and deemed a candidate for cardiac transplantation.

## Introduction

Amyloid cardiomyopathy is the result of amyloid deposition in the myocyte interstitium, causing an increased ventricular myocardial thickness and stiffness, with atrial infiltration, culminating in cardiac dysfunction of restrictive physiology pattern. Amyloid transthyretin cardiomyopathy (ATTR-CM) has two types, namely the hereditary and wild types. The prevalence of hereditary ATTR in the United States is 1 in 100,000 persons, while worldwide, around 50,000 persons are affected, with 80% predominantly having cardiomyopathy and 20% predominantly having polyneuropathy [1,2]. The wild type has a prevalence of 4.15% in males and 1.05% in females in the population ≥75 years, reaching up to 13.9% of males over 85 years [3]. ATTR-CM presents commonly with features of heart failure, with a worsening course. Early diagnosis of the disease through multimodality imaging is helpful to commence treatment. Our vignette describes an interesting case of progressive heart failure symptoms as initial manifestations of transthyretin cardiac amyloidosis, which could advance hastily to end-stage heart failure if not recognized early. Management is focused on stabilizing the transthyretin fibrils in the myocardium, in addition to the guideline-directed medical therapy (GDMT) for heart failure, to prevent the worsening deleterious outcome of untreated transthyretin cardiac amyloidosis.

## Case presentation

A 67-year-old male with a history of hypertension, diabetes, and hyperlipidemia presented with mild to moderate, nonexertional, intermittent pressure-like substernal chest discomfort of four days' duration. He claimed that he tires easily and noted worsening bilateral lower extremity edema for almost six months now. He also endorsed that he can now only ambulate one block due to shortness of breath, which had been progressively worse over the last couple of months, with significant progression from the NYHA (New York Heart Association) functional class II to III. He has also been having episodes of orthopnea and paroxysmal nocturnal dyspnea for the past two months.

He had a history of bilateral carpal tunnel syndrome, status post-release surgery. He had no significant family history of cardiac disease or sudden cardiac death. He is a nonsmoker, nonalcoholic drinker, and denied recreational drug use. He has a temperature of 98°F, blood pressure of 106/70 mmHg, heart rate of 71, respiratory rate of 17, and oxygen saturation of 97% on room air. Physical examination revealed a 2/6 mitral regurgitation murmur and trace bipedal edema. He had no jugular vein distension or crackles.

**Figure 1:**
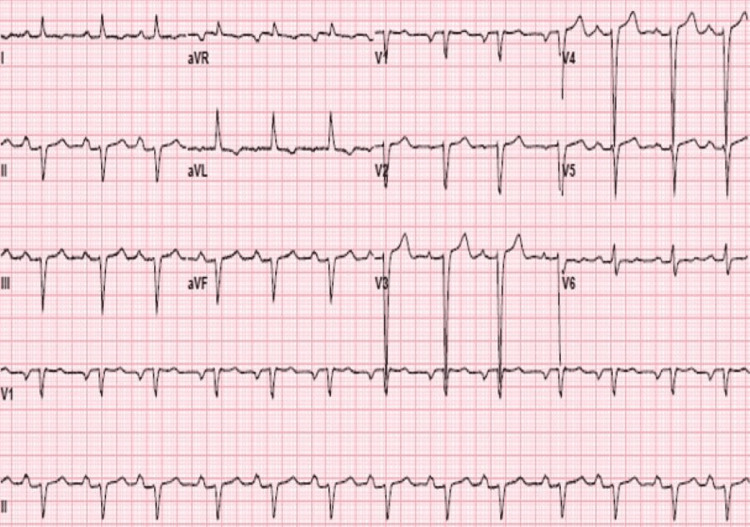
Twelve-lead electrocardiogram revealing normal sinus rhythm, with first-degree atrioventricular block, left axis deviation, poor R wave progression, left atrial enlargement, and premature atrial contractions.

**Figure 2:**
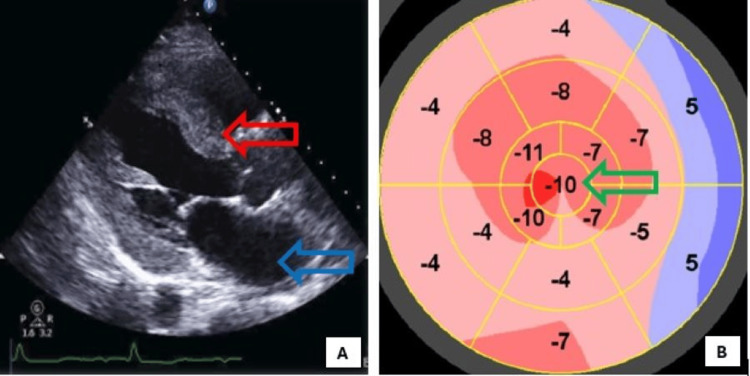
Transthoracic echocardiogram showing left atrial enlargement (blue arrow), left ventricular hypertrophy (red arrow) (A), with diffuse hypokinesia, severe left ventricular systolic dysfunction with an ejection fraction of 10%-15% (on cine images), and reduced global longitudinal strain with some apical sparing (green arrow) (B) in end-stage cardiac amyloidosis.

An electrocardiogram showed normal sinus rhythm with first-degree atrioventricular block, left axis deviation, poor R wave progression, left atrial enlargement, and premature atrial contractions (Figure 1). He had a mildly elevated troponin I that peaked at 0.198 ng/mL. A transthoracic echocardiogram showed biatrial enlargement, left ventricular hypertrophy with diffuse hypokinesia, severe left ventricular systolic dysfunction with an ejection fraction of 10%-15%, abnormal filling pattern suggestive of increased left ventricular filling pressures, and reduced global longitudinal strain with apical sparing (Figure 2). He was then diagnosed with new-onset heart failure with reduced ejection fraction.

**Figure 3:**
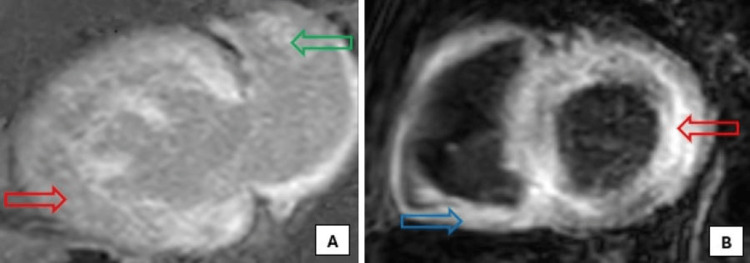
Cardiac magnetic resonance imaging showing global and subendocardial left ventricle (red arrows), right ventricle (blue arrow), and left atrium (green arrow), myocardial fibrosis on delayed gadolinium enhancement due to abnormal contrast kinetics, consistent with cardiac amyloidosis (A - two-chamber view, B - short-axis view).

He had a cardiac catheterization, which revealed nonobstructive disease with less than 30% stenosis in the proximal right coronary artery and left anterior descending artery. He was admitted to the coronary care unit and was placed on GDMT for heart failure and coronary artery disease. During his course, cardiac magnetic resonance imaging was done, which showed global and subendocardial biatrial and biventricular myocardial fibrosis on delayed enhancement due to abnormal contrast kinetics, consistent with cardiac amyloidosis (Figure 3).

**Figure 4:**
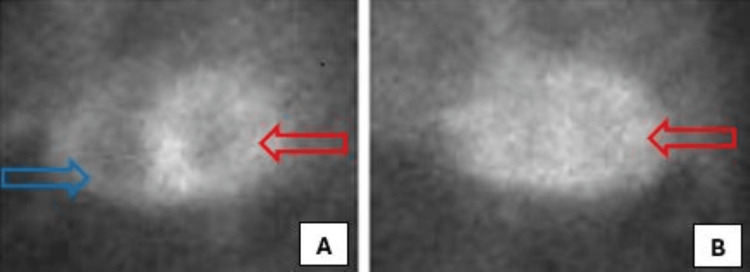
Technetium-99m pyrophosphate scan revealing marked (4+) diffuse increase in tracer concentration throughout the left (red arrows) and right (blue arrow) ventricular myocardium, consistent with diffuse cardiac amyloidosis of the transthyretin type (A - left anterior oblique view, B - lateral view).

**Figure 5:**
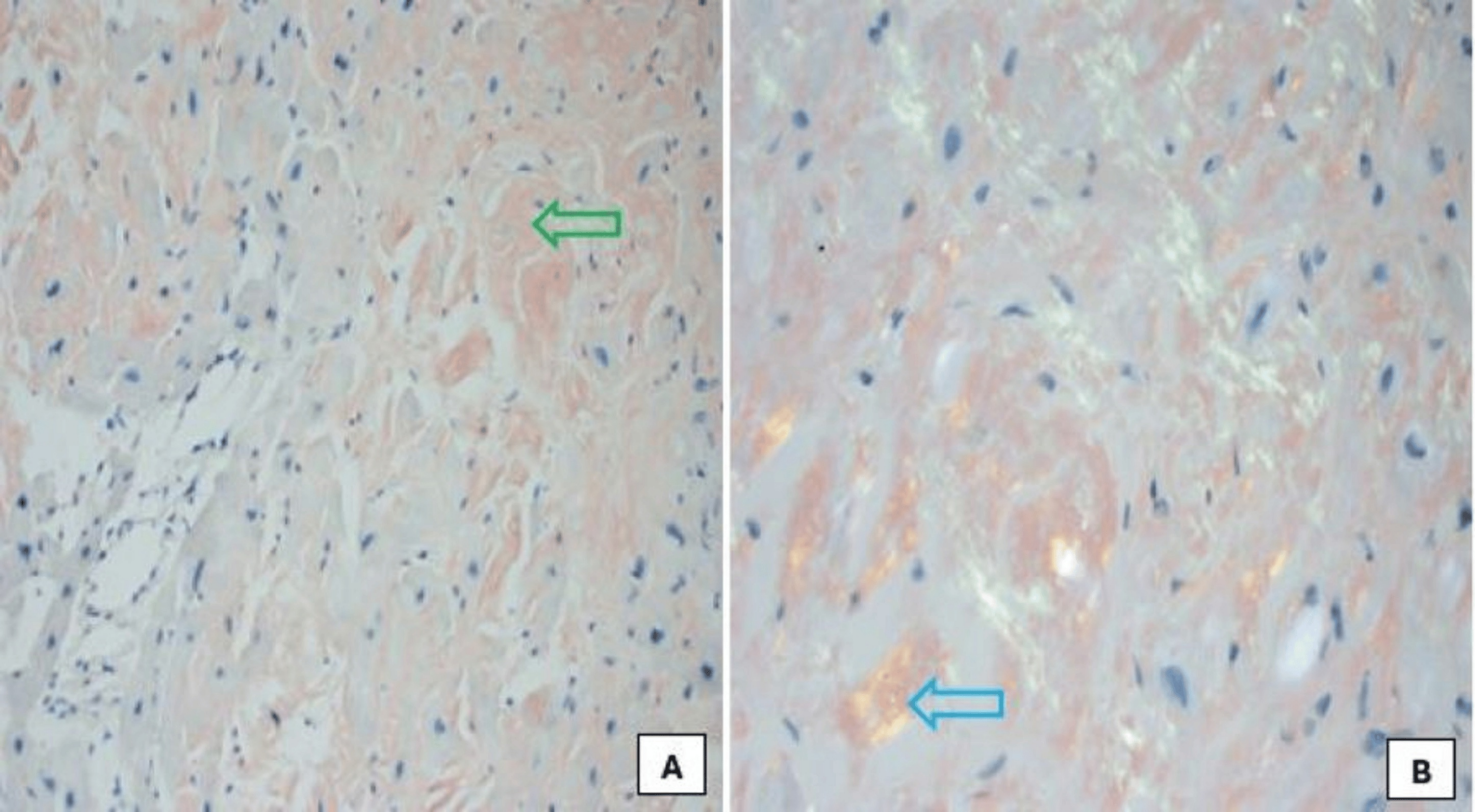
Histological image of cardiomyocytes stained with Congo red, showing amyloid deposits (A - green arrow, x200) and apple-green birefringence on polarized light (B - blue arrow, x400).

A technetium-99m pyrophosphate scan was also done to confirm the diagnosis, which revealed a marked (4+) diffuse increase in tracer concentration throughout the left and right ventricular myocardium with a heart-to-contralateral lung (H/CL) ratio of 1.6, consistent with cardiac amyloidosis of the transthyretin type (Figure 4). He also underwent a cardiac biopsy, which revealed amyloid deposition on Congo red stain, confirmed by apple-green birefringence on polarized light (Figure 5). An amyloid subtyping determined a peptide profile consistent with ATTR deposition.

Genetic testing was done, confirming a mutation in the transthyretin gene (Val122Ile), which strongly suggests hereditary ATTR amyloidosis. Amyloid light-chain (AL) cardiac amyloidosis had been quickly ruled out because of its rapid progressive course, which typically involves chemotherapy, and noted an unremarkable serum-free light-chain, as well as serum and urine immunofixation.

He was started on furosemide 40 mg intravenously twice daily, then transitioned to an oral regimen of 40 mg twice daily, lisinopril 5 mg daily, spironolactone 12.5 mg daily, aspirin 81 mg daily, and atorvastatin 20 mg daily. He was also placed on fluid and salt restriction. He improved significantly during his course in the hospital and was discharged with close surveillance in the cardiology outpatient clinic. He had a regular follow-up in the clinic and has been compliant with his medications.

He was eventually started promptly on Vyndaqel (tafamidis) 80 mg daily, an oral transthyretin stabilizer, which he has been taking for almost two years now. On surveillance in the clinic, he had a significant improvement in his heart failure symptoms and his exercise capacity, which was noted as early as six months while on Vyndaqel (tafamidis). He has not been readmitted to the hospital since he started the oral transthyretin stabilizer in addition to his maintenance heart failure regimen.

Periodic surveillance echocardiogram while on therapy improved his left ventricular systolic function to 35%-40%. He had been referred for cardiac transplantation evaluation even prior to the initiation of Vyndaqel (tafamidis).

## Discussion

Amyloidosis is an infiltrative disease characterized by the extracellular accumulation of fibrillary protein into solitary or several organs. Cardiac amyloidosis is the result of amyloid deposition in the interstitium of cardiomyocytes, which has been an increasingly recognized etiology of restrictive cardiomyopathy and congestive heart failure [2]. There are two types of cardiac amyloidosis, namely AL and ATTR cardiac amyloidosis. AL amyloidosis arises from aggregated AL fibrils, resulting from the overproduction of light-chain kappa or lambda immunoglobulin by the monoclonal plasma cells in the bone marrow. On the other hand, ATTR amyloidosis develops from the aggregation of either native, called senile systemic amyloidosis, or variant TTR protein, referred to as familial amyloidosis, manufactured by the liver of individuals with mutations in the TTR gene, of which more than 100 single nucleotide polymorphisms and 80 confirmed mutations have been elucidated [4]. The mutation ensues destabilization of the stable, soluble tetramer transthyretin protein into misfolded monomers or dimers, creating fibril sheets that can accumulate in the heart and other organs [5]. Senile systemic amyloidosis affects individuals mostly in their seventh decade of life and consistently involves the heart, which is known as the wild-type variant. In contrast, the variant TTR protein that causes familial cardiac amyloidosis affects individuals in their fourth decade of life or later and causes familial amyloid cardiomyopathy, familial amyloid neuropathy, or mixed disease of cardiomyopathy and neuropathy [6].

Both types of amyloidosis involve the heart; however, both carry different prognoses and, therefore, have been targeted by different treatment strategies. Diastolic and systolic dysfunction may be the initial symptoms in individuals with cardiac amyloidosis; however, the concomitant occurrence of various symptoms from different organ systems hinders early consideration of cardiac amyloidosis. Other clinical hints, including bilateral carpal tunnel syndrome, bilateral biceps tendon rupture, and autonomic dysfunction, should prompt screening for cardiac amyloidosis. Conduction system disease is also a frequent manifestation of ATTR cardiac amyloidosis, and only about 46% to 60% of AL patients and about 25% to 40% of patients with ATTR-CM meet true low-voltage electrocardiographic criteria [4]. Amyloid deposition in the vasculature can also contribute to perfusion abnormalities, which at times mimic microvascular angina. The similar clinical features of AL and ATTR cardiac amyloidosis also hinder the ability to differentiate these two entities of cardiac amyloidosis.

Cardiac amyloidosis is characterized by biventricular thickening, along with systolic and diastolic dysfunction, as a component of a systemic infiltrative process [7]. It is highly essential to diagnose the disease early to commence treatment because of its dismal prognosis compared with other etiologies of left ventricular hypertrophy. Albeit endomyocardial biopsy is the definitive modality in the diagnosis of cardiac amyloidosis, it is invasive, expensive, and entails expertise, which can hinder the timely diagnosis, thus, it is rarely required when 99mTc-pyrophosphate scintigraphy confirms ATTR cardiac amyloidosis with absent monoclonal proteins.

Multimodality noninvasive imaging tests are readily available for diagnosis, which explore the distinctive features of the disease. Nuclear medicine modalities and cardiovascular magnetic resonance imaging uncover the pathological features of the disease; however, the utilization of these studies is still constrained [8,9]. Echocardiography is an accessible first-line imaging modality in the investigation of heart disease, including cardiac amyloidosis. It has been a well-established tool in the diagnosis of cardiac amyloidosis because of the distinct features of the disease. The typical echocardiographic findings on echocardiography may provide a hint to consider cardiac amyloidosis in subsets of patients with thick left ventricular walls of unexplained etiology. Besides, it can provide essential information on filling pressures of the left ventricle, as well as hemodynamic data, which are needed in the management of such a disease process. Determination of left ventricular remodeling, diastolic function assessment [10,11], and utilization of deformation-based parameters with typical apical-sparing, with 93% sensitivity and 82% specificity [12,13], have been suggested as echocardiographic indices to distinguish cardiac amyloidosis from other causes of left ventricular hypertrophy. Ejection fraction global longitudinal strain ratio (EFSR) has the best echocardiographic accuracy in identifying cardiac amyloidosis and is independent of the underlying amyloidosis type (AL or ATTR) [7]. Cardiac magnetic resonance imaging shows global and subendocardial late gadolinium enhancement [14] with sensitivity of 86%-88% and specificity of 86%-90% [15,16]. The emergence of noncontrast T1 mapping allowed more accurate detection of cardiac amyloidosis and conforms appropriately with indicators of systolic and diastolic dysfunction [17]. Echocardiography and cardiac magnetic resonance imaging cannot differentiate between light-chain amyloidosis and transthyretin amyloidosis. However, the utilization of bone scintigraphy tracers facilitated the initial detection of transthyretin amyloidosis [18], which distinguishes the two types of amyloidosis [19,20]. The 99mTc-pyrophosphate planar and single-photon emission computed tomography cardiac imaging has 97% sensitivity and 100% specificity for establishing transthyretin cardiac amyloidosis, compared with light-chain cardiac amyloidosis, as evidenced by a significantly higher semiquantitative visual score of tracer uptake and by quantitative analysis [20].

## Conclusions

Our vignette emphasized the early utilization of different cardiac imaging modalities to unravel the diagnosis of transthyretin cardiac amyloidosis. Clinical hints include symptoms of heart failure, low voltage complexes on electrocardiogram despite evidence of left ventricular hypertrophy, bilateral carpal tunnel syndrome, bilateral biceps tendon rupture, and autonomic dysfunction, which should prompt screening for cardiac amyloidosis. Amyloid cardiomyopathy presents commonly with features of heart failure, and early diagnosis of the disease through multimodality imaging is helpful to institute timely and appropriate treatment. ATTR-CM should be explored as an etiology of heart failure symptoms, and therapy should be geared towards initiation of GDMT in addition to the promising benefits of the new oral transthyretin stabilizing agents. Other treatment options should be considered, including gene silencers (patisiran, inotersen, and vutrisiran) in patients with ATTR-associated polyneuropathy.
